# Epidemiological, clinical and therapeutic profiles of mantle cell lymphoma cared for in a Moroccan center: a review of 14 cases

**DOI:** 10.11604/pamj.2024.47.111.40405

**Published:** 2024-03-06

**Authors:** Ouadii Abakarim, Adil Mansouri, Abdelaziz Hebbezni, Imane Boujguenna, Fatima Ezzahra Lahlimi, Illias Tazi

**Affiliations:** 1 Department of Clinical Hematology and Bone Marrow Transplantation, Mohammed VI University Hospital, Faculty of Medicine and Pharmacy, Cadi Ayyad University, Marrakesh, Morocco,; 2 Clinical Research Unit, Mohammed VI University Hospital, Marrakesh, Morocco,; 3 Department of Radiology, Mohammed VI University Hospital, Faculty of Medicine and Pharmacy, Cadi Ayyad University, Marrakesh, Morocco,; 4 Pathology, Faculty of Medicine and Pharmacy, Ibn Zohr University, Guelmim, Morocco

**Keywords:** Mantle cell lymphoma, immunochemotherapy, Morocco, case series

## Abstract

Mantle cell lymphoma (MCL) accounts for 3-10% of non-Hodgkin's lymphomas (NHL). We identified 14 patients with mantle cell lymphoma, with an average number of 3.5 new cases/year. A male predominance was observed with a sex ratio equal to 6. The average age of our patients was 64.4±14.1 years, with an average diagnostic delay of 6.57 months. Regarding the clinical presentation, adenopathy was the most reported physical sign (78.6%) followed by B symptoms (57.1%). Disseminated stages were the most frequent in our series: stages IV (78.5%) and III (7.1%) versus stages I (0%) and II (7.1%). The extra-ganglionic localizations observed were hepatic 5 cases (31.1%), pulmonary 04 cases (25%), medullary 4 cases (25%), pleural 2 cases (12.5%) and prostate 1 case (6.2%). All diagnosed cases are mantle cell lymphomas, of which 12 cases (85.7%) are classical and 2 cases (14.3%) indolent. The high-risk group is, according to international prognostic index (MIPI) MCL prognostic score, the most represented in our series: 0-3 = 6 cases (42.9%), 6-11 = 8 cases (57.1%). The therapeutic protocol chosen 1^st^ line: 9 patients treated with R-DHAP, three with R-CHOP, one with DHAOX and one with R-CVP. Second line: two patients treated with R-DHAP, one after R-CHOP and the other after R-CVP. Two patients received autologous hematopoietic stem cell transplant at the end of the treatment. The evolution was marked by the death of 7 patients, 3 lost to follow-up and 4 still followed. Additionally, the study highlights characteristics and treatment patterns of mantle cell lymphoma, emphasizing its predominance in males, delayed diagnosis, frequent dissemination, and high-risk classification, with chemotherapy as the primary treatment modality and a challenging prognosis contributing to a comprehensive understanding of mantle cell lymphoma presentation and management.

## Introduction

Mantle cell lymphoma (MCL) accounts for 3-10% of non-Hodgkin's lymphomas (NHL) [1]. The 2016 World Health Organization (WHO) classifications clearly identify two variants of MCL: a classical variant and an indolent variant [2]. Mantle cell lymphoma is characterized by the presence of a t(11;14) chromosomal translocation (q13;32), which is responsible for cyclin D1 overexpression inducing cell cycle dysregulation [2,3]. In most patients, the diagnosis is made at an advanced stage of the disease with a median survival of no more than 4 years [4,5]. Extra-nodes involvement is often present [6]. The current first-line treatment is based on six cycles of rituximab, cisplatin, cytarabine and dexamethasone (R-DHAP), followed, in young patients, by an autologous hematopoietic stem cell transplant (AHSCT) [7]. Our aim is to describe the clinical and therapeutic characteristics of mantle cell lymphoma managed in the hematology department at the Mohammed VI University Hospital of Marrakesh.

## Methods

**Study design and setting:** this observational study, categorized as a case series study, involved conducting a retrospective chart review at the hematology department of the Mohammed VI University Hospital of Marrakesh.

**Study population:** patients diagnosed with MCL and treated at our department from January 2018 to December 2021 were included. Only patients with histologically and immunohistochemical confirmed MCL were considered, while those with doubtful diagnoses were excluded.

**Data collection:** to identify patients, we reviewed individual medical records accessed through hospitalization, day hospital, and consultation registers. This process was facilitated by using a predefined paper form. We extracted various information including demographic details such as age and sex, follow-up details, disease type specifying histology and relevant criteria, therapeutic management data, and severity variables.

**Definitions:** mantle cell lymphoma was defined as a lymphoid neoplasm characterized by a typical morphology and/or a typical immunophenotype. Diagnosis relied on adhering to the diagnostic criteria outlined in relevant histological and immunohistochemical guidelines [2].

**Statistical analysis:** it was conducted using IBM SPSS Statistics (version 16.0, IBM Corp, Armonk, NY). Descriptive statistics were employed to analyze the case series data, with categorical variables presented as frequencies and percentages, and quantitative variables summarized using median and interquartile range.

**Ethical considerations:** the study adhered to the ethical principles of the Declaration of Helsinki, ensuring the anonymity and confidentiality of all extracted patient data. In accordance with Moroccan regulations, no consent was required as the study did not involve any interventions.

## Results

**Figure 1:**
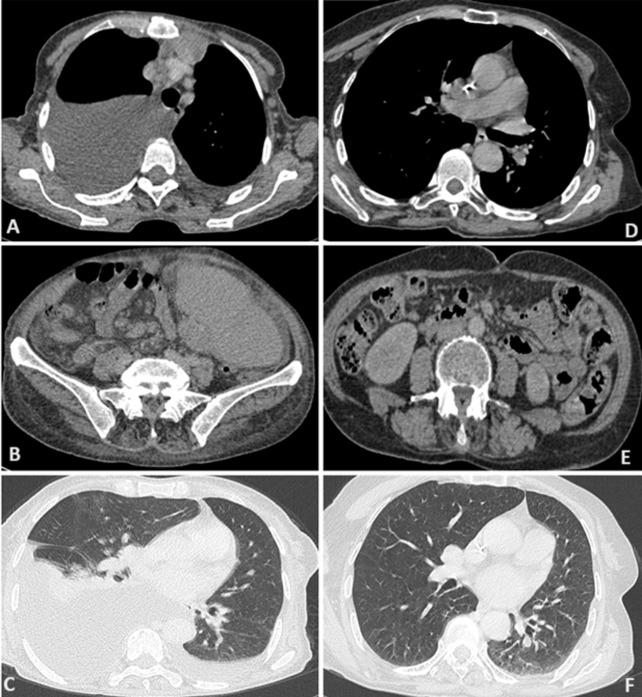
computed tomography scan of patients with stage VI mantle cell lymphoma before (A, B, C) and after (D, E, F) chemotherapy, demonstrating regression of tumor burden evident across thoracic, abdominal, and pelvic regions

**Figure 2:**
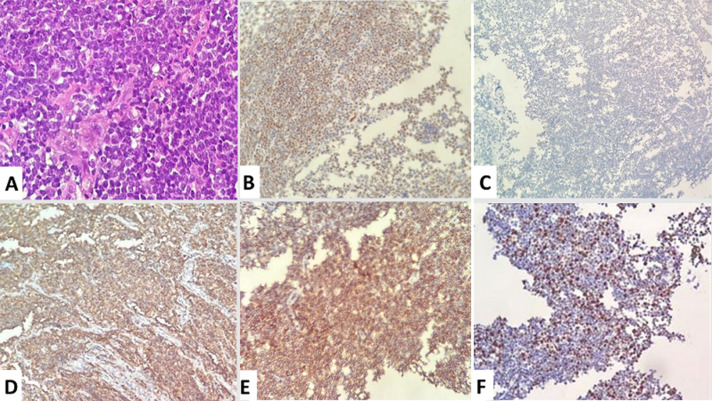
histopathological images of patients with mantle cell lymphoma: A) lymphomatous proliferation with medium-sized, monotonous cells and cleaved nuclei (x40 magnification); immunohistochemistry revealing positivity for markers: B) CD10; C) cyclin D1; D) CD20; E) CD5; F) Ki67 at 50%

Between 2018 and 2021, a total of 14 patients with mantle cell lymphoma were identified in the hematology department at the Mohammed VI University Hospital of Marrakesh. With an average number of 3.5 new cases/year. A male predominance was noted with a sex ratio (male/female) at 6. The average age of our patients was 64.4±14.1 years, with an average diagnostic delay of 6.57 months (Table 1). Regarding the clinical presentation, adenopathy was the most reported physical sign (78.6%) followed by B symptoms (57.1%). All patients benefited from a cervico-thoraco-abdomino-pelvic CT scan, which revealed the tumor syndrome (Figure 1 A,B,C). Disseminated stages were the most frequent in our series: stages IV (78.5%) and III (7.1%) versus stages I (0%) and II (7.1%). The extra-nodes localizations observed were hepatic five cases (31.1%), pulmonary four cases (25%), medullary four cases (25%), pleural two cases (12.5%) and prostate one case (6.2%) (Table 2). All diagnosed cases are mantle cell lymphomas (Figure 2), of which 12 cases (85.7%) are classical and two cases (14.3%) indolent. The high-risk group is, according to MIPI prognostic score, the most represented in our series: 0-3 = 6(42.9%), 6-11 = 8 (57.1%) (Table 3). The therapeutic protocol chosen in our series were: i) first line: Nine patients treated with R-DHAP, three with rituximab, cyclophosphamide, doxorubicin, vincristine and prednisone (R-CHOP), one with rituximab, dexamethasone, cytarabine and oxaliplatin (R-DHAOX), one with rituximab, cyclophosphamide, vincristine and prednisone (R-CVP); ii) second line: two patients treated with R-DHAP, one after R-CHOP and the other after R-CVP. Two patients received an autologous hematopoietic stem cell transplant at the end of the treatment. The evolution was marked by the death of seven patients, three lost to follow-up and four still followed with good improvement (Figure 1 D,E,F).

**Table 1:** epidemiological characteristics of patients

	Number of cases (n)	Percentage (%)
**Age, median(min-max)**	65.5 (38.0-85.0)	
**Gender**		
Male	12	85.7
Female	2	14.3
**Family situation**		
Single	1	7.1
Married	13	92.9
**Health insurance**		
Yes	13	92.9
No	1	7.1
**Level of study**		
Illiterate	9	64.3
Primary	4	28.6
Secondary	1	7.1
**Professional activity**		
Yes	6	42.9
No	8	57.1
**Antecedent**		
Tobacco	1	7.1
Diabetes	1	7.1
Upper tension	1	7.1

**Table 2:** clinical and immunohistochemical characteristics of patients

	Number of cases (n)	Percentage (%)
**Reason for consultation**		
Node	6	42.9
Abdominal distension	1	7.1
Abdominal mass	3	21.4
Pruritus	1	7.1
Anemic syndrome	1	7.1
**Diagnostic time (months)**	5.5 (1-12)	
**Clinical examination**		
Lymphadenopathy	11	78.6
Splenomegaly	6	42.9
Fever	8	57.1
Weight loss	8	57.1
Tonsillar hypertrophy	1	7.1
**Primitive localization**		
Axillary	5	35.7
Cervical	8	57.1
Tonsil	1	7.1
Supraclavicular	1	7.1
Prostate	1	7.1
Submental	1	7.1
**Immunohistochemistry**	14	100
CD20 +	14	100
CD23 +	2	15.4
Cyclin D1 +	12	85.7
BLC6 +	2	28.6
Ki67 +	9	90.0
BCL2 +	8	57.1
CD5+	11	84.6

**Table 3:** distribution of patients by grade staging and simplified mantle cell lymphoma pronostic index risk category

	Number of cases (n)	Percentage (%)
**Ann Arbor Staging**		
I	0	0
II	2	14.3
III	1	7.1
IV	11	78.6
**sMIPI**		
High-risk	9	64.3
Intermediate-risk	0	0
Low-risk	5	35.7
sMIPI: simplified mantle cell lymphoma pronostic index		

## Discussion

Between 2018 and 2021, the Mohammed VI University Hospital of Marrakesh identified 14 patients with mantle cell lymphoma, our findings underscore the clinical characteristics, treatment patterns, and outcomes of mantle cell lymphoma patients. These findings emphasize the importance of early detection, comprehensive treatment strategies, and continued monitoring to improve patient outcomes in this challenging disease. Mantle cell lymphoma, originally described as a centrocytic lymphoma in the Kiel classification [8], is defined in the WHO classification as a mature B-cell neoplasm and has distinguished a subtype of lymphoma, termed aggressive lymphoma [2]. Nevertheless, some recent studies have identified patients presenting a more indolent course [9-11]. Two cases were indolent form in our series. MCL occurs mainly in older men (sex ratio ≥2: 1) with a median age of about 60 years (range, 29 to 85 years) [12]. In the same way, a male predominance was noted in our studies, with a mean age of 64.4±14.1 years (range 38-85 years). Patients are commonly in stage III/IV disease with extensive lymphadenopathy, splenomegaly, blood and bone marrow involvement [4]. Disseminated stages III/VI were the most frequent in our series (85.6%). Other extra-nodes localizations were mainly in the gastrointestinal tract, the liver, or Waldeyer's ring [6]. We observed in our patients, hepatic (31.1%), pulmonary (25%), medullary (25%), pleural (12.5%) and prostate (6.2%) involvement. The diagnosis is histological and is made on the basis of a lymph node biopsy, tissue, bone marrow or blood phenotype that shows the typical morphology of small to medium-sized monomorphic lymphoid cells with irregular nuclear contours [2]. Indeed, MCLs express the neoplasm markers of mature B cells, namely CD19, CD20, CD22, CD79-A, and Cyclin D1. These cells are also CD5+, CD10-, CD23-, CD43+ and BCL6- [2]. All our patients showed small cell lymphoid infiltration as histological morphology with CD20+ (100%), CD23+ (41.7%), CD5+ (84.6%), Cyclin D1+ (85.7%), BCL2+ (57.1%), CD10- (84.6%), CD23- (41.7%), and BCL6- (71.4%) on immunohistochemistry. Overexpression of SOX11 as a transcription factor has been reported as a diagnostic marker of MCL, its absence is indicative of the indolent form [3]. Other features such as a high Ki-67 mitotic index or p53 mutations and p16 deletions are tightly linked to more aggressive MCL subtypes such as blastoid variants [13].

The Ki-67 index was highly positive in half of our patients. A few cases with MCL are negative for cyclin D1 expression, this was the case in two of our patients. It is a molecular variant of MCL, as they share a similar genetic profile as well as similar clinical features [14]. Numerous clinical, histological, and laboratory parameters have been studied to assess the potential for predicting the heterogeneous evolution of MCL patients. The most useful clinical prognostic model is the simplified international prognostic index MCL (MIPI), combining age, performance status, lactate dehydrogenase and lymphocyte count. The use of the Ki-67 mitotic index may provide further prognostic value [15]. Eight of our cases were high risk and six were low risk. There is no standard first-line treatment for MCL and still incurable. Several regimens provide longer response times [16]. In our study, Therapeutic protocols included various chemotherapy regimens as first-line treatments, such as R-DHAP, R-CHOP, R-DHAOX, and R-CVP. Second-line treatments, including R-DHAP, were administered to two patients. Considering the poor prognosis and that standard therapy does not seem to cure MCL patients, abstention from therapy for indolent, low MIPI or elderly forms should be considered [17]. For young patients aged ≤65-70 years requiring therapeutic management, fit for intensive chemotherapy, the initial treatment of MCL is based on R-DHAP alone or in combination with R-CHOP followed by consolidation AHSCT with or without rituximab-based maintenance therapy alone which has demonstrated good outcomes [18-20]. For patients over 70 years of age with co-morbidities requiring therapeutic management, not eligible for Autologous haematopoietic stem cell transplantation (AHSCT), the choice of protocol varies between R-CHOP with rituximab maintenance, bendamustine and rituximab (BR) ± rituximab maintenance, and R-CVP [21-23]. Unfit patients, not eligible for chemotherapy, the therapeutic choice is based on rituximab with lenalidomide, rituximab with ibrutinib or rituximab alone [24,25].

These protocols are also recommended for relapsed/refractory MCL [26,27]. We have adopted the same therapeutic strategy for our patients. Allogeneic hematopoietic stem cell transplantation (allo-HCT) after myeloablative conditioning has little place in the treatment of MCL [28]. Many therapies like venetoclax, an oral bcl-2 inhibitor, and chimeric antigen receptor (CAR) T cells are currently being studied [29-31]. The findings from this study have several important implications for clinical practice and research. Firstly, the male predominance and the relatively advanced stage of presentation highlight the need for increased awareness and targeted screening efforts for mantle cell lymphoma, particularly among older male populations. Early detection is crucial for improving treatment outcomes and potentially reducing diagnostic delays, which could lead to better overall survival rates. Secondly, the therapeutic choices reflected in the study, including various chemotherapy regimens and stem cell transplantation, provide insights into current treatment protocols for mantle cell lymphoma. These findings underscore the importance of personalized treatment approaches based on disease characteristics, patient factors, and risk stratification. Continued research into novel treatment modalities and targeted therapies is warranted to further improve outcomes and reduce treatment-related toxicities.

## Conclusion

Mantle cell lymphoma, an aggressive lymphoma with limited survival, is diagnosed based on histology, immunohistochemistry, and cytogenetics, often presenting with extra-nodal localizations more frequently than other lymphomas. Current treatment primarily involves polyimmuno-chemotherapy followed by AHSCT, particularly in relatively young patients with good general condition. However, therapeutic advancements in the future are expected to stem from a deeper understanding of oncogenesis, leading to the identification of new therapeutic targets. This study reinforces the significance of early detection, personalized treatment approaches, comprehensive supportive care, and enhanced patient engagement in managing mantle cell lymphoma. The insights gleaned from this research are invaluable for clinicians, researchers, and policymakers alike, aiming to optimize care delivery and enhance outcomes for patients grappling with this challenging hematological malignancy.

### What is known about this topic

- *Mantle cell lymphoma (MCL) is a rare incurable subtype of non-Hodgkin lymphoma;*
- *The 2016 World Health Organization updated classification describes 2 major subtypes, classical and leukemic non-nodal MCL, each with unique molecular features and clinical presentations;*
- *Despite an improved understanding of biology and the development of effective therapeutic strategies resulting in improved survival, patients continue to have a poor prognosis overall*.

### What this study adds

- *Clinical and therapeutic characteristics of mantle cell lymphoma managed in Moroccan hospital;*
- *We have shown the different treatment approaches according to different situations;*
- *Many therapies like venetoclax, an oral bcl-2 inhibitor, and chimeric antigen receptor (CAR) T cells are currently being studied*.
